# Modulation of Tumor Immune Microenvironment and Prognostic Value of Ferroptosis-Related Genes, and Candidate Target Drugs in Glioblastoma Multiforme

**DOI:** 10.3389/fphar.2022.898679

**Published:** 2022-04-28

**Authors:** Xudong Zhang, Shengnan Jin, Xin Shi, Shengyu Liu, Kunhang Li, Guojun Liu, Shiyu Zhong, Tao Liu, Lishuai Li, Shanwei Tao, Qingqing Zhai, Nan Bao, Lijie Ren, Ying Wu, Yijun Bao

**Affiliations:** ^1^ Department of Neurosurgery, The Fourth Hospital of China Medical University, Shenyang, China; ^2^ Department of Developmental Cell Biology, China Medical University, Shenyang, China; ^3^ School of Maths and Information Science, Shangdong Technology and Business University, Yantai, China; ^4^ Business School, All Saints Campus, Manchester Metropolitan University, Manchester, United Kingdom; ^5^ Department of Neurosurgery, Luoyang Central Hospital Affiliated to Zhengzhou University, Luoyang, China; ^6^ School of Management, Shanghai University, Baoshan, China; ^7^ College of Medicine and Biological Information Engineering, Northeastern University, Shenyang, China; ^8^ Health Science Center, Neurology Department of Shenzhen Second People’s Hospital, The First Affiliated Hospital of Shenzhen University, Shenzhen, China; ^9^ Phase I Clinical Trails Center, The First Affiliated Hospital of China Medical University, Shenyang, China

**Keywords:** ferroptosis, glioblastoma multiforme, IDH1, prognosis, multi-omics analysis

## Abstract

Glioblastoma multiforme (GBM) is the most common type of malignant brain tumor, among which IDH1-wild type GBM has a poor prognosis. Recent studies have shown that ferroptosis-related genes (FRGs) are correlated with the development and progression of cancer. In GBM, the role of FRGs associated with IDH1 status as biological indicators and therapeutic targets remains to be clarified. Ten of FRGs (STEAP3, HSPB1, MAP1LC3A, SOCS1, LOX, CAPG, CP, GDF15, CDKN1A, and CD44) associated with IDH1 status in GBM were identified as key genes through screening by survival analysis and Random Forest using The Cancer Genome Atlas (TCGA) datasets, and the protein expressions of key genes were verified. Transwell and qPCR results showed that ferroptosis promoted the migration of glioblastoma cells and affected the expression of key genes. Our study established the ferroptosis-related prognostic model for GBM patients based on ten key genes by a different modeling method from previous study, the GSVA algorithm. Further, we took the methods of functional enrichment analysis, clinical characteristics, immune cell infiltration, immunomodulator, ESTIMATE and single nucleotide variant (SNV) analysis to study the molecular mechanisms of prognostic model and key genes. The results showed that ten key genes were strongly associated with immune-related factors and were significantly involved in the p53 signaling pathway, senescence and autophagy in cancer, and in the negative regulation of protein kinase activity. Moreover, potential therapeutic drugs were identified by Virtual Screening and Molecular Docking. Our study indicated that the novel ferrotosis-related prognostic model for GBM patients and key genes possessed the prognostic and therapeutic values.

## 1 Introduction

Glioblastoma multiforme (GBM) is the most common malignant primary intracranial tumor with a poor prognosis despite the existence of therapeutic modalities including surgery, radiotherapy, and chemotherapy. The median survival is only 14–16 months (Ricard et al., 2012; Cancer Genome Atlas Research et al., 2015; Jiang et al., 2016; Chai et al., 2019). According to the World Health Organization (WHO) central nervous system (CNS) cancer classification, GBM can be divided into isocitrate dehydrogenase (IDH)-wild and IDH-mutant types, and IDH-wild type GBM has a relatively poorer prognosis (Yan et al., 2009; Louis et al., 2016). IDH1 mutation is one of the most common and earliest detected genetic alterations in diffuse gliomas, and evidence supports this mutation as a driver of glioma development (Agnihotri et al., 2014).

Ferroptosis is a novel iron-dependent, non-apoptotic form of cell death that kills cells through lipid peroxidation injury occurring on the cell membrane. Although the physiological role of ferroptosis remains to be elucidated, there is ample evidence that ferroptosis plays a very important role in organic diseases such as heart, liver, kidney, and brain (Fang et al., 2019; Jiang et al., 2021), particularly in the treatment of cancer by targeted key ferroptosis-related genes (FRGs) and pathways (Jiang et al., 2021).

In recent years, high-throughput sequencing technologies and genetic databases have been widely used in cancer diagnosis and prognosis studies. Although the role of FRGs in GBM has been initially investigated, few prognosis-related genes have been screened in previous literature (Zhu et al., 2021). The prognostic effectiveness and therapeutic performance of differential expressions of FRGs in IDH1-wild and IDH1-mutant GBM have not been investigated.

Therefore, our study aimed to find FRGs associated with IDH1 status in GBM, which are promising prognostic indicators and therapeutic targets for GBM. We analyzed the sequencing data of GBM patients in TCGA databases by bioinformatics, machine learning algorithm and multi-omics to identify FRGs associated with prognosis in GBM patients as key genes, and the protein expression of the key genes was validated. Ferroptosis was identified by Transwell and qPCR experiments to be associated with the migration ability of glioblastoma cells and affected the expression of key genes. We then established and validated the ferroptosis-related prognostic model for GBM patients. We also studied the possible regulatory mechanisms in terms of the impacts of model and key genes on cancer immunity, biological function, gene mutation and clinical characteristics. Furthermore, we identified potential therapeutic compounds through Virtual screening and Molecular docking.

## 2 Materials and Methods

### 2.1 Data Collection

TCGA-GBM transcriptome expression profile data and genomic mutation data were downloaded through xena (Goldman et al., 2020), 166 TCGA-GBM tumor samples and five normal tissue samples were obtained. 290 FRGs were obtained by merging the datasets from literature (Liang et al., 2020; Zhuo et al., 2020), FerrDb (Zhou and Bao, 2020), MSigDB (Liberzon et al., 2015), and genecards (Stelzer et al., 2016). In addition, immunomodulators, including immunoinhibitors, immunostimulators and MHC molecules, were downloaded from the TISIDB database (Ru et al., 2019).

### 2.2 Identification of Key Genes and Survival Analysis

PCA scatter plot was analyzed using the R package ggplot2 (Wickham, 2016) and screening of DEG using R package limma (Ritchie et al., 2015). Genes with *p* < 0.05 and |log2 fold change (FC)| > 0.5 were considered as DEGs. Further genes screening using the R package randomForest (Liaw and Wiener, 2002), and genes with negative horizontal coordinate values (%IncMSE <0) were filtered, and WIPI1 and SOCS1 were deleted, but SOCS1 was strongly associated with GBM and retained. Ten key genes were obtained finally. Kaplan-Meier curves were plotted, and *p*-value < 0.05 was deemed to be a significant difference between high- and low-risk groups. Immunohistochemical results for key genes were obtained from the Human Protein Atlas (HPA) database (Uhlén et al., 2005). The sample sizes for each group were much larger than three. Antibody staining in the annotated cell types in the current human tissue is reported as not detected, low, medium, or high. The score is based on the staining intensity and fraction of stained cells, therefore the staining scores in different groups are comparable.

### 2.3 Biological Functional Analysis and Correlation Analysis of Clinical Characteristics

GO and pathway functional enrichment analysis of ten key genes were performed using R package cluster profiler (Yu et al., 2012). The correlation between each gene expression with the GBM clinical characteristics (IDH1 status, gender, and risk level) were analyzed and visualized by drawing mosaic plots with the R package mosaic (Pruim et al., 2017).

### 2.4 Cell Culture and Migration Assay

The human glioblastoma cell line U87MG was obtained from the Cell Resource Centre of Peking Union Medical College and U251MG from American Type Culture Collection. Cells were cultured in DMEM medium supplemented with 10% FBS and placed in a standard constant temperature CO_2_ incubator (5% CO_2_, 37°C). The Transwell system (24-well, 8 μm pore size polycarbonate membrane) was for *in vitro* migration assays. U251MG and U87MG cells were pretreated with ferroptosis activator erastin (10 μM) or control solvent for 24 h. Finally the cells attached to the lower surface of the filter membrane were fixed with 4% PFA and then stained with crystal violet. The migrated cells were photographed with a light microscope and counted using ImageJ software. The qPCR primer sequences were all obtained from Primerbank and synthesized by Sangon Biotech (Spandidos et al., 2009), and PCR primer sequences was shown in Supplementary Table S1. All experiments were repeated more than three times.

### 2.5 Construction of Prognostic Model and Nomogram

Enrichment scores based on key gene sets were calculated for each sample using the GSVA algorithm (Hänzelmann et al., 2013) and KM curve was plotted. Survival scatterplot was analyzed using the R package ggplot2 and heatmap using the R package pheatmap, showing the expression of the key genes both in high- and low-risk groups. GSVA score was combined with the clinical indicators (age, sex, and radiation) for univariate and multifactor Cox regression analysis, respectively, and the nomogram and calibration curves were drawn.

### 2.6 Correlation Analysis of Immune Cell Infiltration, Immunomodulators and ESTIMATE Score

Calculation of immune cell infiltration levels for each sample of TCGA-GBM was performed using CIBERSORT website. Wilcox test was used to analyze the difference in immune cell infiltration between high- and low-risk groups and was considered significant with *p*-value < 0.001. Correlations between the gene expression and different immune cells were calculated and considered significant with *p*-value < 0.001 and |r| > 0.2. The stromal score and immune score were calculated for each sample using the ESTIMATE package (Yoshihara et al., 2013).

### 2.7 Single Nucleotide Variant Analysis of Key Genes

The maf data of varscan of TCGA-GBM were downloaded from the TCGA database. The key gene mutations were analyzed, and the SNV distribution was plotted using the R package maftools (Mayakonda et al., 2018).

### 2.8 Virtual Screening and Molecular Docking

The structural information of corresponding compounds was downloaded from DrugBank database (Wishart et al., 2018) and screened according to Lipinski’s rule (hydrogen bond acceptor ≤10, hydrogen bond donor ≤5, rotatable bond ≤10, the logarithmic value of lipid-water partition coefficient ≤5, the molecular weight of 180–480, and polar surface area ≤140). Finally, 5,464 small molecule compounds were obtained. The spatial structure information of the key gene-encoded proteins was searched in the PDB database to find the relevant structural information of CAPG, CP and CD44 (Berman et al., 2000). The corresponding PDB files 6IGX, 4ENZ, and 4PZ3 were downloaded, and the approximate docking box range was determined based on the ligand information therein. We repaired the missing residues using modeller (Eswar et al., 2007). After setting other relevant parameters, we used autodock-vina to dock with the small molecule compounds separately and performed interaction force analysis using Ligplus. PyMol demonstrated docking conformations.

## 3 Results

### 3.1 Identification of Key Genes

#### 3.1.1 Differentially Expressed Ferroptosis-Related Genes associated with IDH1 Status

**FIGURE 1:**
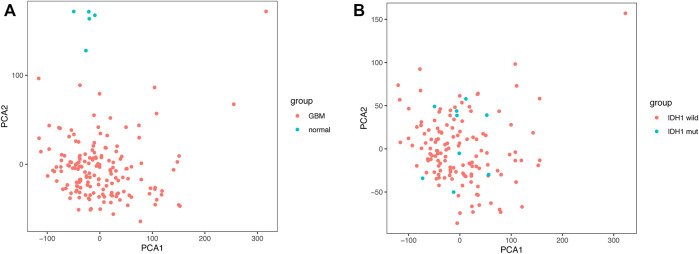
Principal Component Analysis **(A)** GBM vs normal brain tissue **(B)** IDH1 wild vs. IDH1 mutant.

**FIGURE 2:**
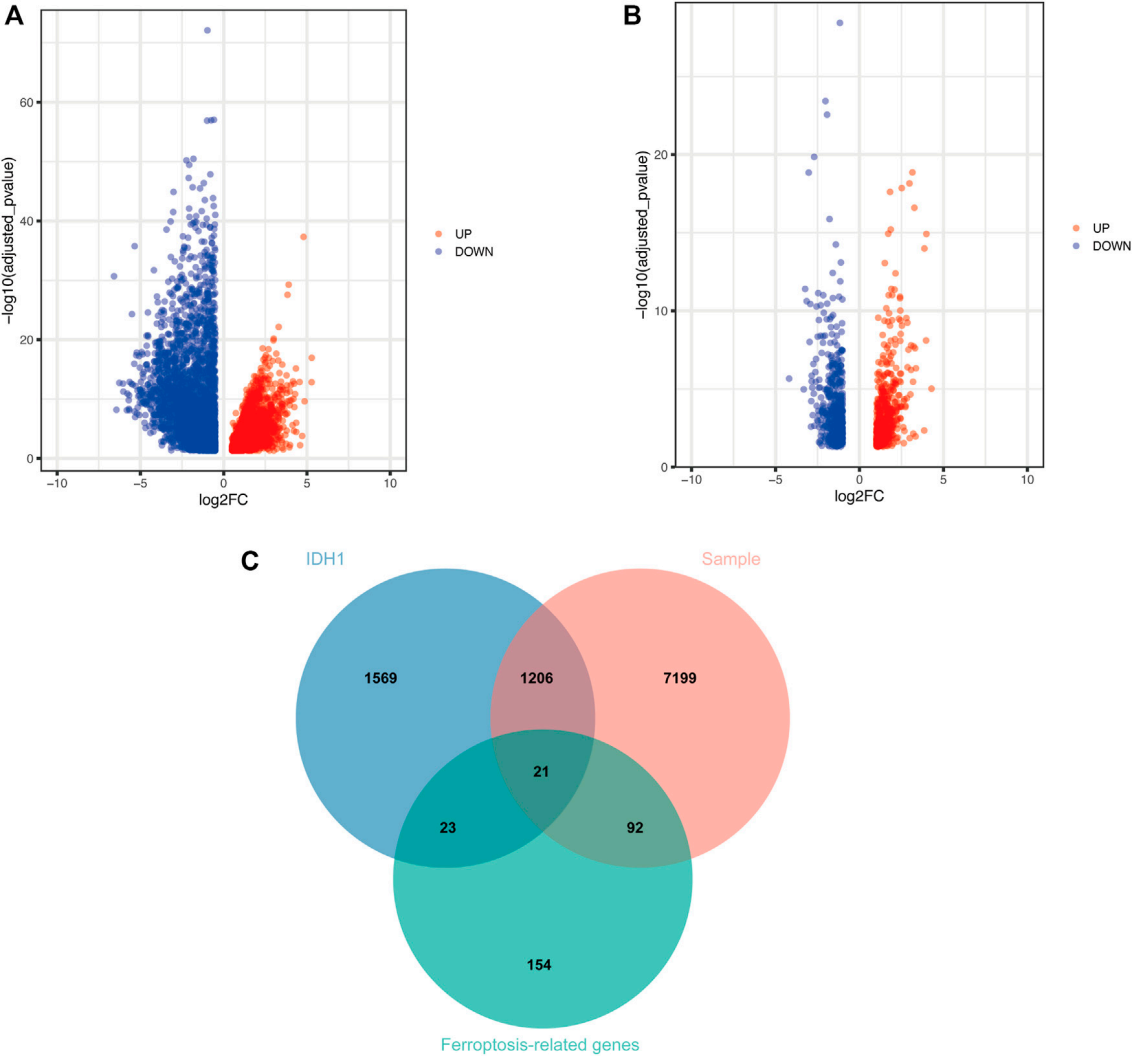
Screening of DEGs **(A)** Volcano plot demonstrates DEGs of GBM vs. normal brain tissue **(B)** Volcano plot shows DEGs of IDH1 wild type vs. mutant type **(C)** Wayne plot indicates shared DEGs.

Given the importance of IDH1 status for the prognosis of GBM patients, we searched for genes related to IDH1 status in GBM patients. The PCA scatter plots of TCGA-GBM expression profile data combined with clinical data were divided into: A) GBM versus normal brain tissue, and B) IDH1 wild versus IDH1 mutant in Figure 1. Screening of differentially expressed genes (DEGs) using GBM versus normal brain tissue expression profile data identified 8,518 DEGs, of which 4,680 were down-regulated, and 3,838 up-regulated (Figure 2A). Similarly, 2,819 DEGs were found in the screening of IDH1 wild type versus mutant type, among 2,819 DEGs, 1771 were down-regulated and the rest of them were up-regulated (Figure 2B). The shared 1,227 DEGs (Figure 2C) were intersected with the collected 290 ferroptosis-related genes (Supplementary Table S2). Consequently, 21 FRGs with significantly differential expressions were obtained.

#### 3.1.2 Ferroptosis-Related Genes associated with Prognosis

**FIGURE 3:**
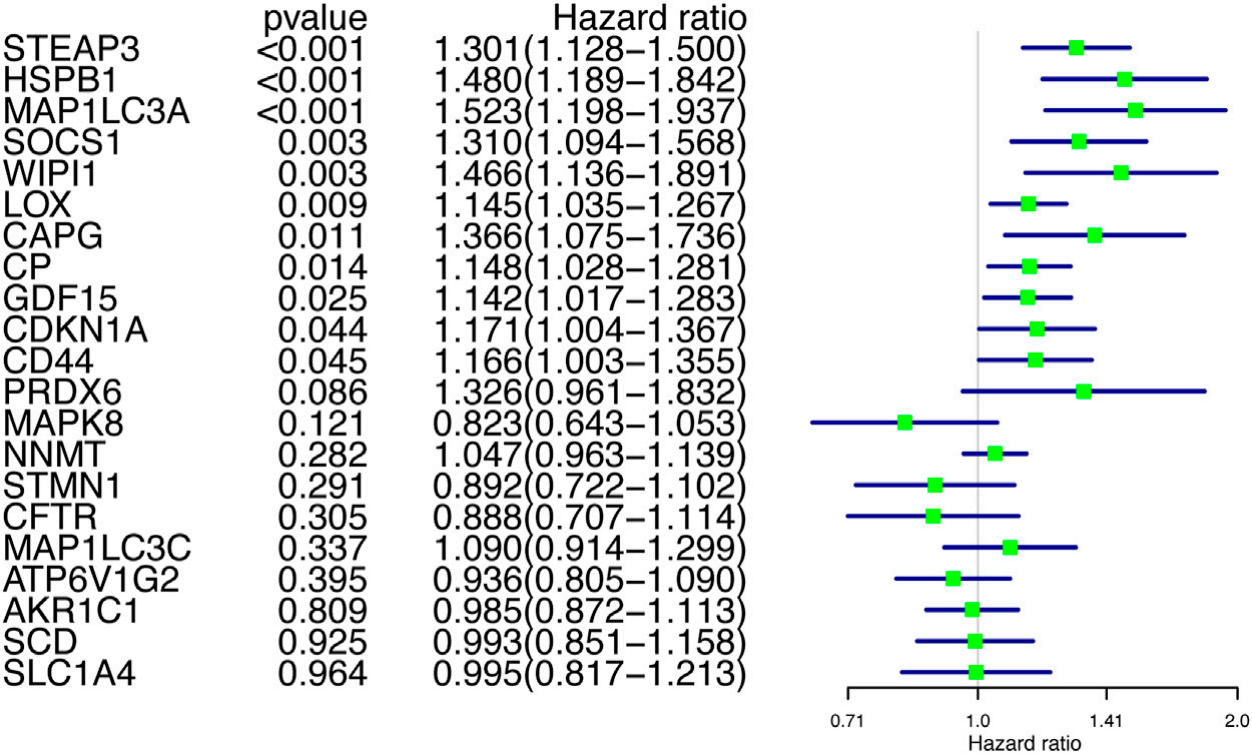
Univariate Cox regression screening for prognosis-related genes.

**FIGURE 4:**
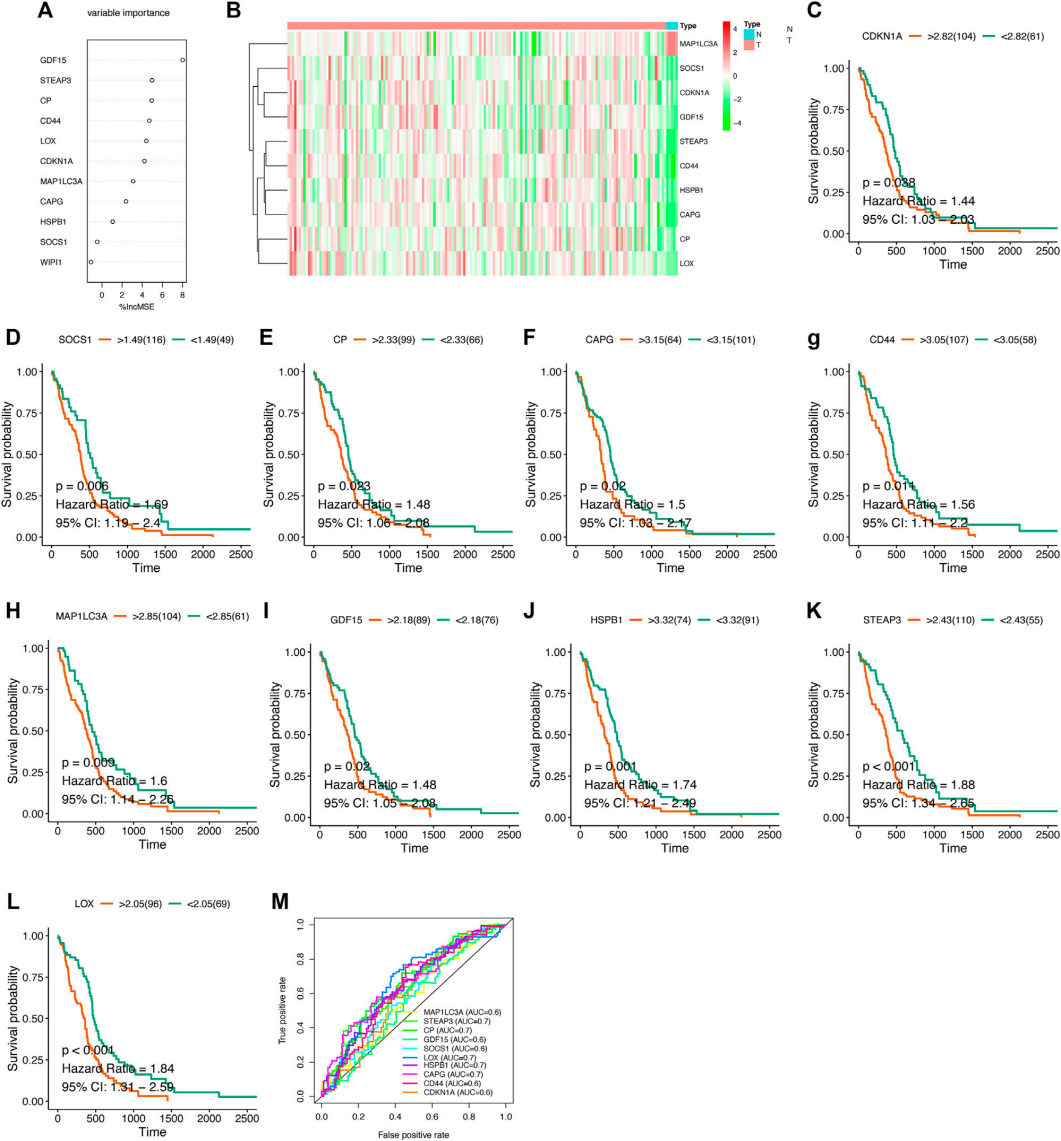
Survival analysis of key genes **(A)** Random Forest screening for ten key genes **(B)** Heat map of expression of key genes in different groups **(C–M)** Kaplan-Meier survival curve and ROC curve.

After obtaining the differentially expressed FRGs, we wanted to verify whether these DEGs were associated with patient prognosis, which would have great clinical significance. So we further screened these FRGs for prognosis-related genes. Because the prognosis of GBM patients is usually poor, the identification of genes that indicate poor prognosis may facilitate the discovery of new therapeutic targets. Using Cox proportions for univariate survival analysis, 11 FRGs associated with prognosis (*p* < 0.05) were found and all of them indicated poor prognosis (HR > 1) (Figure 3). The gene WIPI1 was excluded by applying Random Forest to continue the screening (Figure 4A), then ten key genes (STEAP3, HSPB1, MAP1LC3A, SOCS1, LOX, CAPG, CP, GDF15, CDKN1A, and CD44) were screened and the heat map of expression was plotted in GBM versus normal brain tissue groups. MAP1LC3A was the only gene that high-expressed significantly in the normal brain tissue group (Figure 4B). According to genecards, the expression of MAP1LC3A was indeed suppressed in many tumor cell lines, suggesting that it may be involved in carcinogenesis (Stelzer et al., 2016). Subsequently, all ten key genes were divided into two groups based on the expression levels, and survival and prognosis were assessed by the Kaplan-Meier (KM) survival curves. All ten key genes (including MAP1LC3A) were identified to be at significant risk (*p* < 0.05), and higher expression was associated with poorer prognosis (Figure 4C to 4L). The results of multi-gene ROC curve analysis showed that the area under the curve (AUC) is greater than 0.6 for all genes. The AUCs of STEAP3, CP, LOX, HSPB1, and CAPG were greater than 0.7. These results indicated that these ten key FRGs had good predictive performances (Figure 4M).

### 3.2 Protein Expression of Key Genes

**FIGURE 5:**
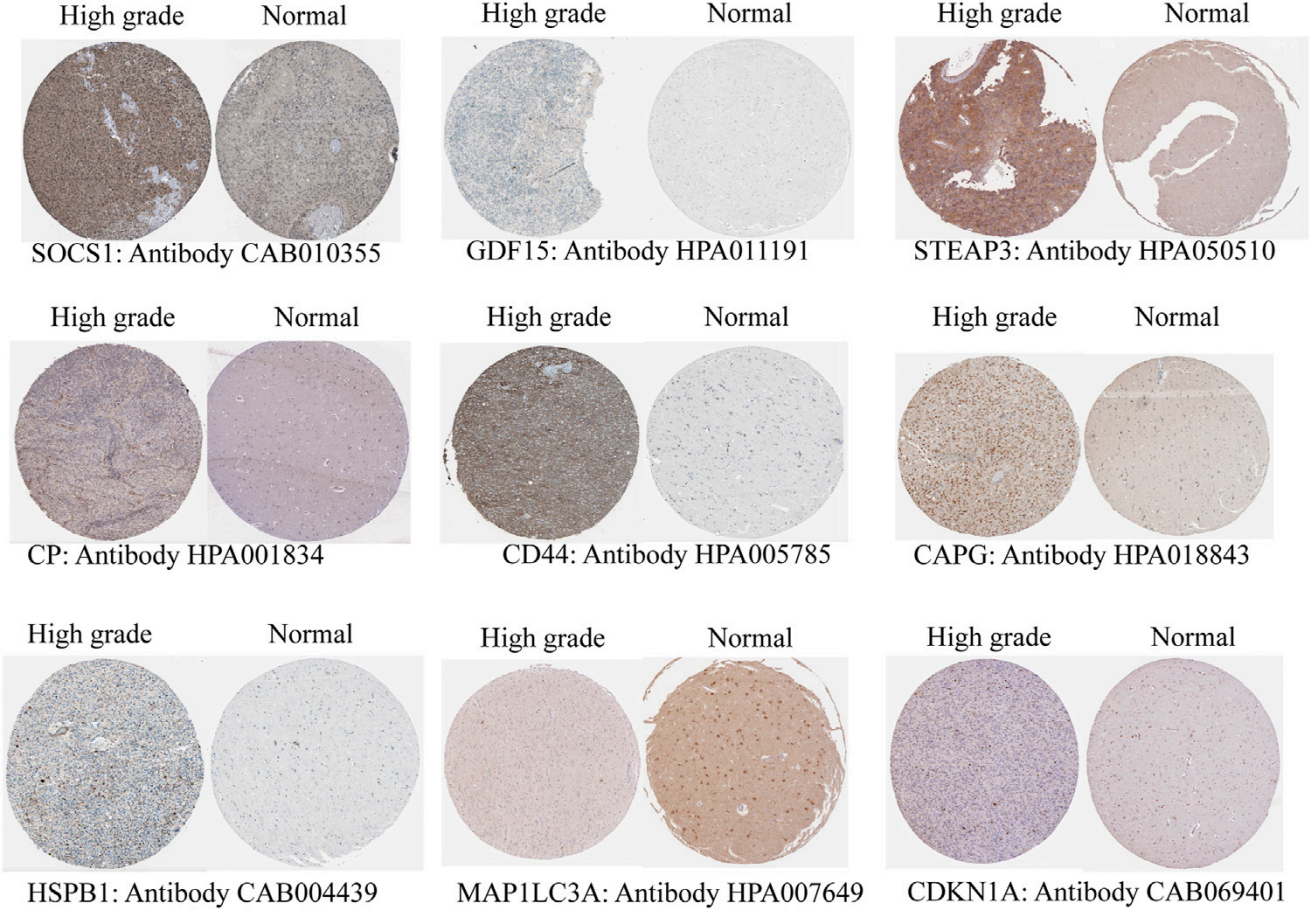
Validation of protein expression by immunohistochemistry of the key genes from the Human Protein Atlas (HPA) database.

To verify different protein expression encoded by the key genes in normal brain tissues and high-grade gliomas, immunohistochemistry analysis was obtained from the Human Protein Atlas (HPA) database (Uhlén et al., 2005). Nine of these key genes were included in the HPA database. STEAP3, HSPB1, SOCS1, CAPG, CP, GDF15, CDKN1A, and CD44 were highly expressed in high-grade gliomas compared with the normal brain tissue, and MAP1LC3A was highly expressed in the normal brain tissue (Figure 5). It was verified that the results of HPA are consistent with the results of our above transcriptome analysis.

### 3.3 Association of Ferroptosis With Cell Migration and Expression of Key Genes

**FIGURE 6:**
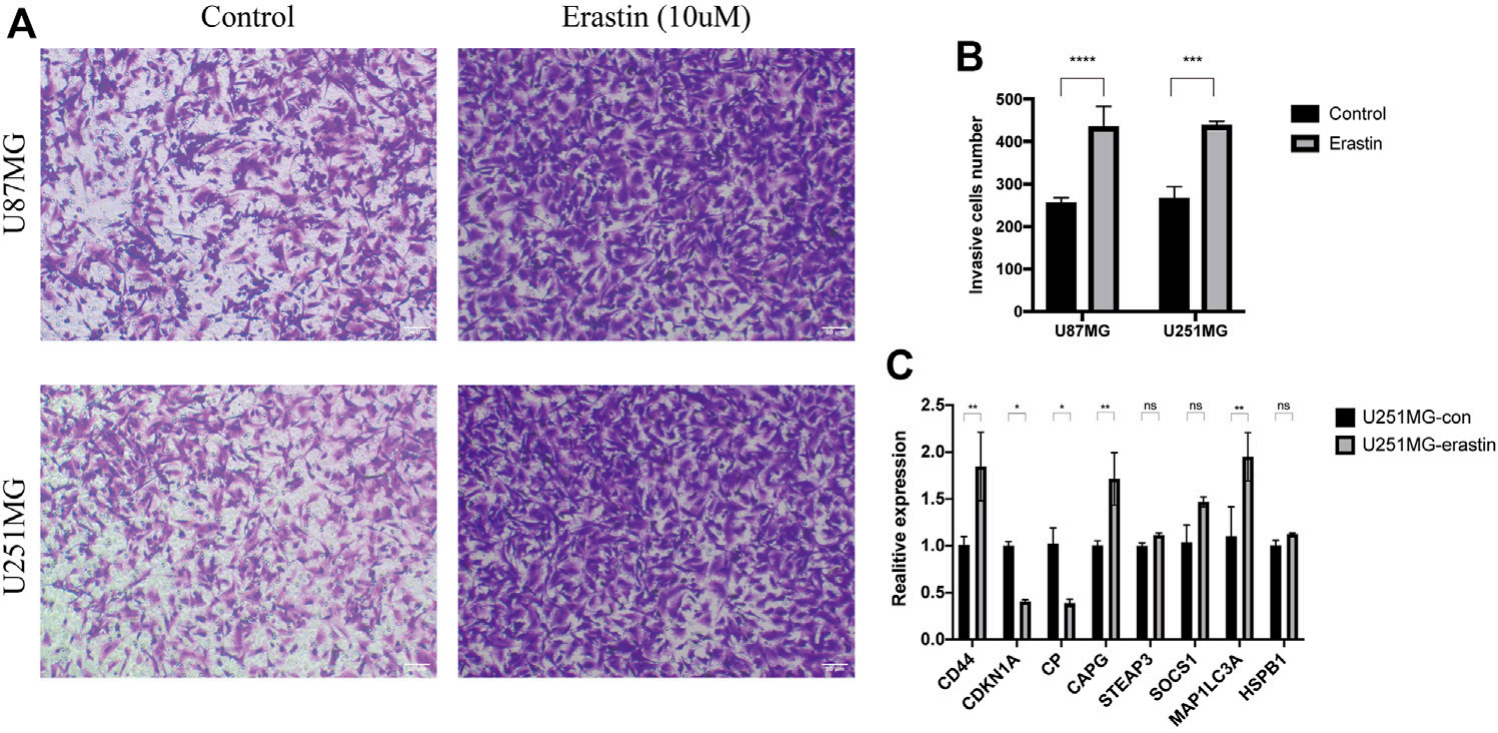
Ferroptosis enhances glioblastoma cell migration **(A–B)** Light microscopic images and analysis of their data showed enhanced migratory ability of U251MG and U87MG cells treated with erastin (10 μm) compared to control **(C)** qPCR analysis showed that the expression of key genes is altered in the erastin (10 μm)-treated group compared to the control cells.

To identify the relationship between ferroptosis and glioblastoma cell migration, we measured the migratory ability of glioblastoma cell lines (U251MG and U87MG) treated with the ferroptosis activator erastin using the transwell migration assay. The migratory ability of erastin-treated U251MG and U87MG cells was increased compared to control treatment (Figures 6A,B). The expression of several key genes was also examined by qPCR, which revealed significant changes in the expression of CD44, CDKN1A, CP, CAPG, and MAP1LC3A in erastin-treated U251MG and U87MG cells, with the upregulation expression of CD44, CAPG, and MAP1LC3A and the down-regulation of CDKN1A and CP (Figure 6C). These results suggest that ferroptosis enhances the migratory ability of glioblastoma cells and alters the expression of these key genes with poor prognosis.

### 3.4 Construction of Prognostic Model

#### 3.4.1 Prognostic Modeling by GSVA

**FIGURE 7:**
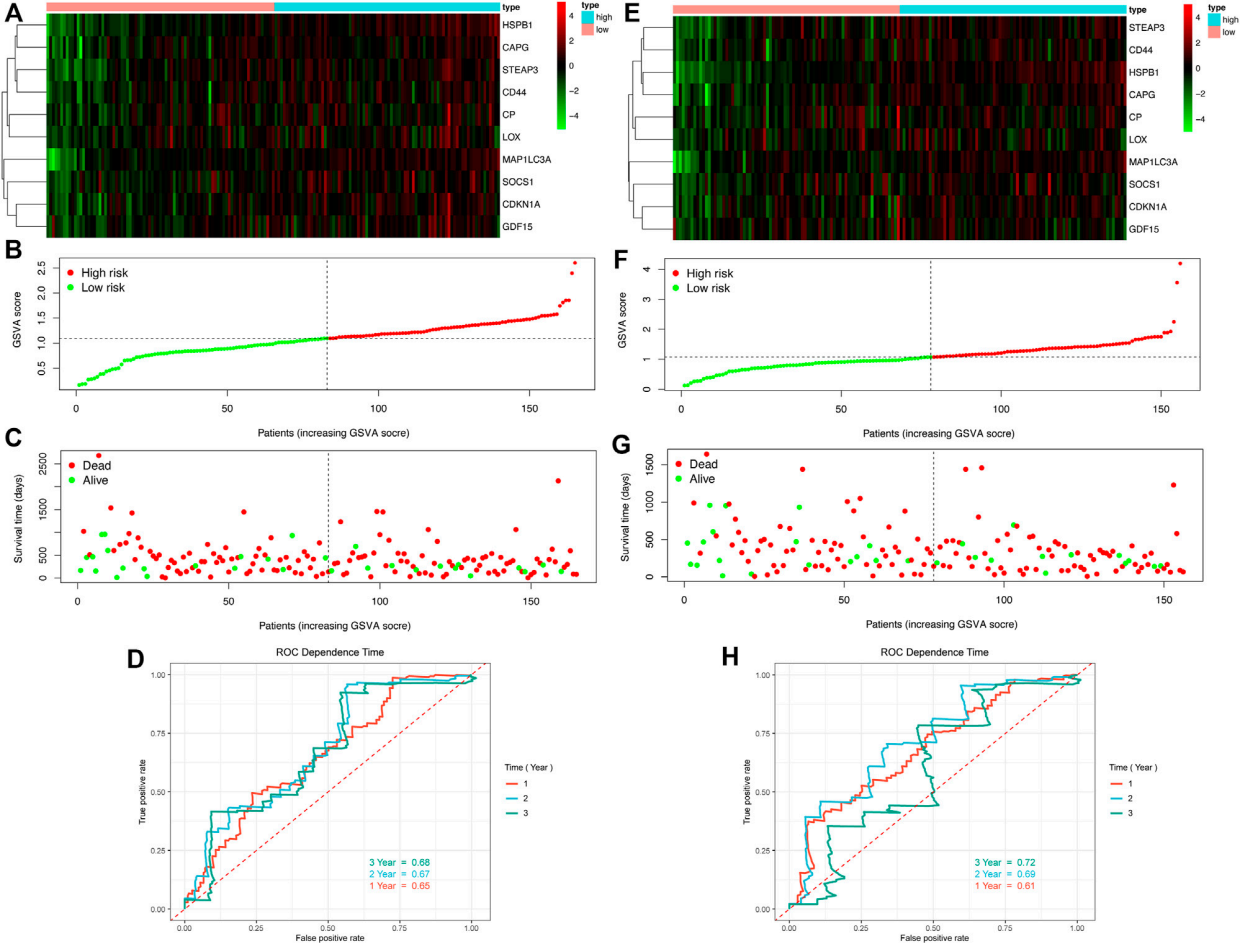
Growth of GSVA score of GBM patients is associated with increased mortality and decreased survival time **(A–D)** OS **(E–H)** RFS **(A,E)** Heat maps of key genes expression profiles **(B–C,F–G)** Distribution of risk scores, patient survival times and status **(D,H)** ROC curves.

Following the confirmation of the prognosis-related single FRG, we constructed a prognostic model by integrating these ten genes, and the GSVA algorithm was used to calculate an enrichment score for each sample based on the key gene set, i.e., the GSVA score. Patients were divided into high- and low-risk groups based on the median GSVA score in overall survival (Figures 7A–D) and relapse-free survival (Figures 7E–H). Key gene heat maps were plotted separately (Figures 7A,E). Risk score, survival time and status of TCGA cohorts in OS were shown in Figures 7B,C, and those in RFS were illustrated in Figures 7F,G. The analysis showed that the mortality rose significantly with the increase of the GSVA score, and the ROC curve showed that the AUCs were all around 0.7, indicating good survival prediction of the model at one-, two- and three-year (Figures 7D,H).

#### 3.4.2 Model Validation and Nomogram

**FIGURE 8:**
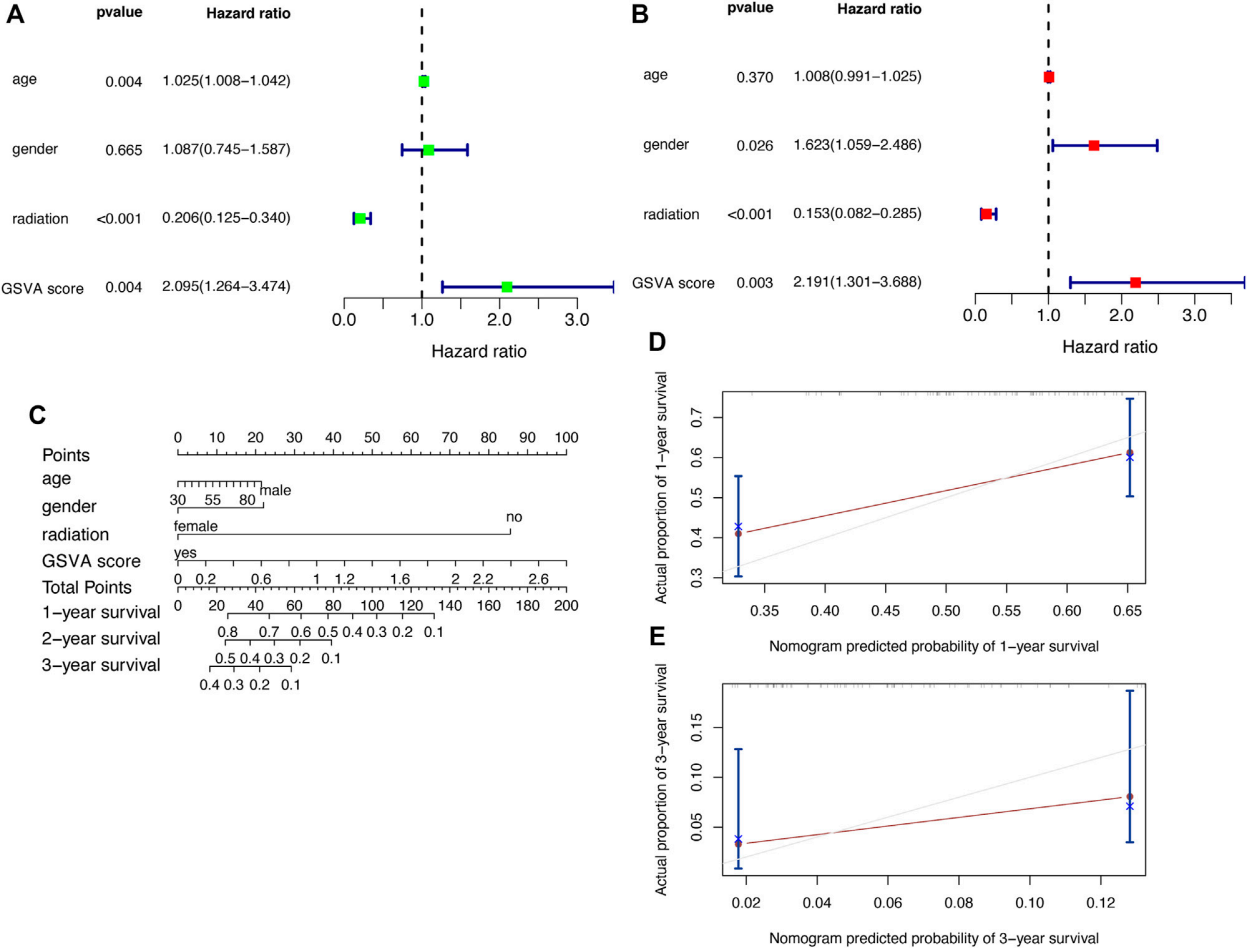
Validation of the prognostic model with clinical factors **(A)** Univariate Cox regression analysis **(B)** Multivariate Cox regression analysis **(C)** Nomogram **(D,E)** Calibration curves.

After obtaining the above prognostic model, we need to verify its predictive power. We performed univariate (Figure 8A) and multivariate Cox analysis (Figure 8B), and the results showed that the GSVA score was an independent prognostic factor for GBM. To assist the clinicial decision-making process, we combined the GSVA score with clinical indicators (age, gender, and radiation) to construct a nomogram (Figure 8C), and the nomogram can predict the survival rate of GBM patients at one-, two- and three-year. The calibration curves (Figures Figure8D,E) indicate that this nomogram has strong predictive function.

### 3.5 Immune Microenvironment Analysis

#### 3.5.1 Immune Cell Infiltration

**FIGURE 9:**
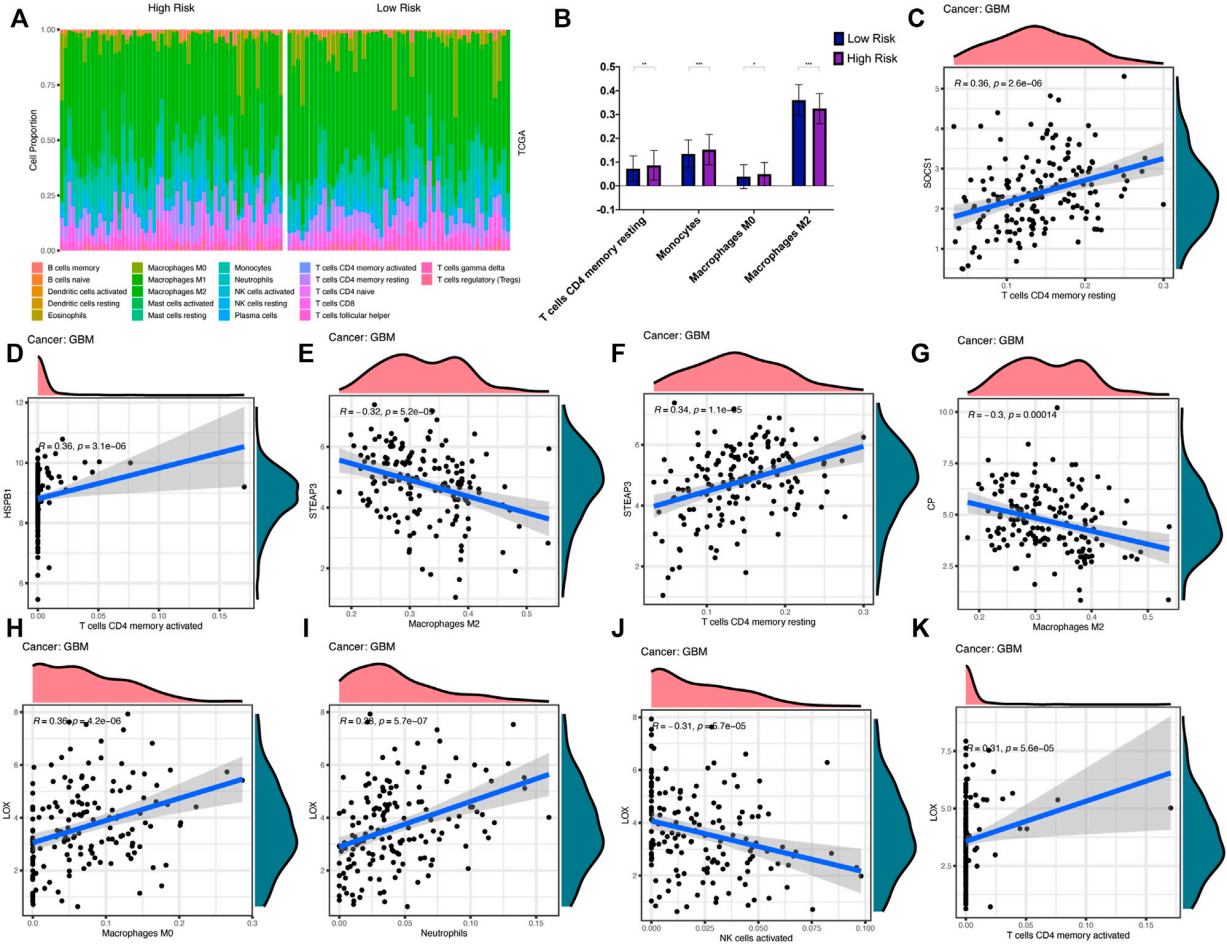
Immune cell infiltration analysis **(A,B)** Infiltration differences of 22 immune cells in high- and low-risk groups in TCGA-GBM **(C–K)** Correlation curves show that infiltration levels of six immune cells are significantly correlated with key genes expression levels.

We analysed the molecular mechanisms of these ten key genes from an immunological perspective. Tumor tissue contains not only tumor cells, but also immune cells. The immune cells that infiltrate tumors can profoundly affect the tumor development and anti-cancer therapy. Therefore, the quantification of immune cells is of extraordinary significance. We assessed the correlation between the prognostic model and the level of immune cell infiltration. The infiltration levels of 22 tumor immune cells in the TCGA-GBM datasets were calculated using the CIBERSORT website, and the differences of immune cell infiltration in the high- and low-risk groups in both datasets were demonstrated (Figures 9A,B). Correlation analysis showed that the infiltration levels of six immune cells were significantly correlated (|r| > 0.3, *p* < 0.05) with some key genes (Figures 9C–K). In particular, STEAP3 (R = −0.32, *p* = 0.000052) and CP (R = −0.3, *p* = 0.00014) were significantly negatively correlated with macrophages M2. LOX (R = −0.31, *p* = 0.000057) was significantly negatively correlated with NK cells activated. Therefore, these key genes were related to the development and prognosis of GBM.

#### 3.5.2 Immunomodulation

**FIGURE 10:**
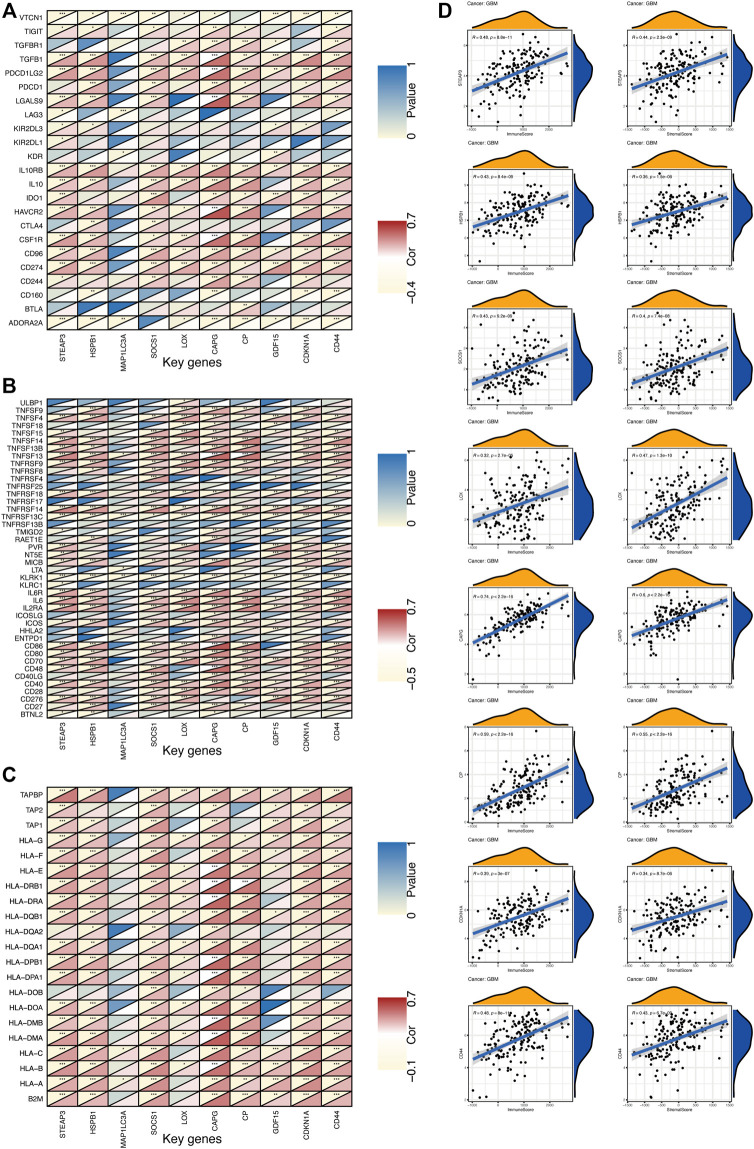
The expression of key genes is correlated with GBM immunity **(A)** Heat map represents the correlation between immunoinhibitor and key gene expression in GBM. For each pair of genes, the triangles at the top left are colored to indicate the *p* values; the triangles at the bottom right are colored to indicate the Spearman correlation coefficients. *p < 0.05; **p < 0.01; ***p < 0.001 **(B)** Heat map represents the correlation between immunostimulator and key gene expression in GBM **(C)** Heat map represents the correlation between MHC molecules and key gene expression in GBM **(D)** The correlation between key genes and ESTIMATE scores in GBM.

In addition, the correlation between key gene expression and immunomodulators was also investigated. As shown in Figure 10, 23 types of immunoinhibitor have been analyzed (Figure 10A). Except for MAP1LC3A with a poor correlation, the remaining genes showed a strong correlation with immunoinhibitor, especially CAPG expression was significantly positively correlated with most of the immunoinhibitor. The correlation analysis of 42 immunostimulator (Figure 10B) showed that MAP1LC3A expression showed weak correlation with immunostimulator, while CAPG and CP showed significantly positive correlation. As shown in Figure 10C, 21 types of major histocompatibility complex (MHC) were analyzed. Human leukocyte antigen (HLA) is the expression product of the human MHC, which is the most complicated polymorphic system known in the human body (Norman et al., 2017). Notably, MHC is closely related to the immune response, immune regulation and the generation of certain pathological states in the body. CAPG unsurprisingly showed an extremely strong positive correlation. ESTIMATE is a tool for predicting tumor purity and the presence of infiltrating stromal and immune cells in the tumor (Yoshihara et al., 2013). The ESTIMATE algorithm generates four final scores: the stromal score (indicating the presence of stromal cells in the tumor tissue), the immune score (indicating the infiltration of immune cells in the tumor tissue), the ESTIMATE score, and the tumor purity. The results of ESTIMATE are summarized in Figure 10D. Remarkably, eight key genes (STEAP3, HSPB1, SOCS1, LOX, CAPG, CP, CDKN1A, and CD44) showed significant positive correlations with stromal score and immune score.

### 3.6 Functional Enrichment Analysis

**FIGURE 11:**
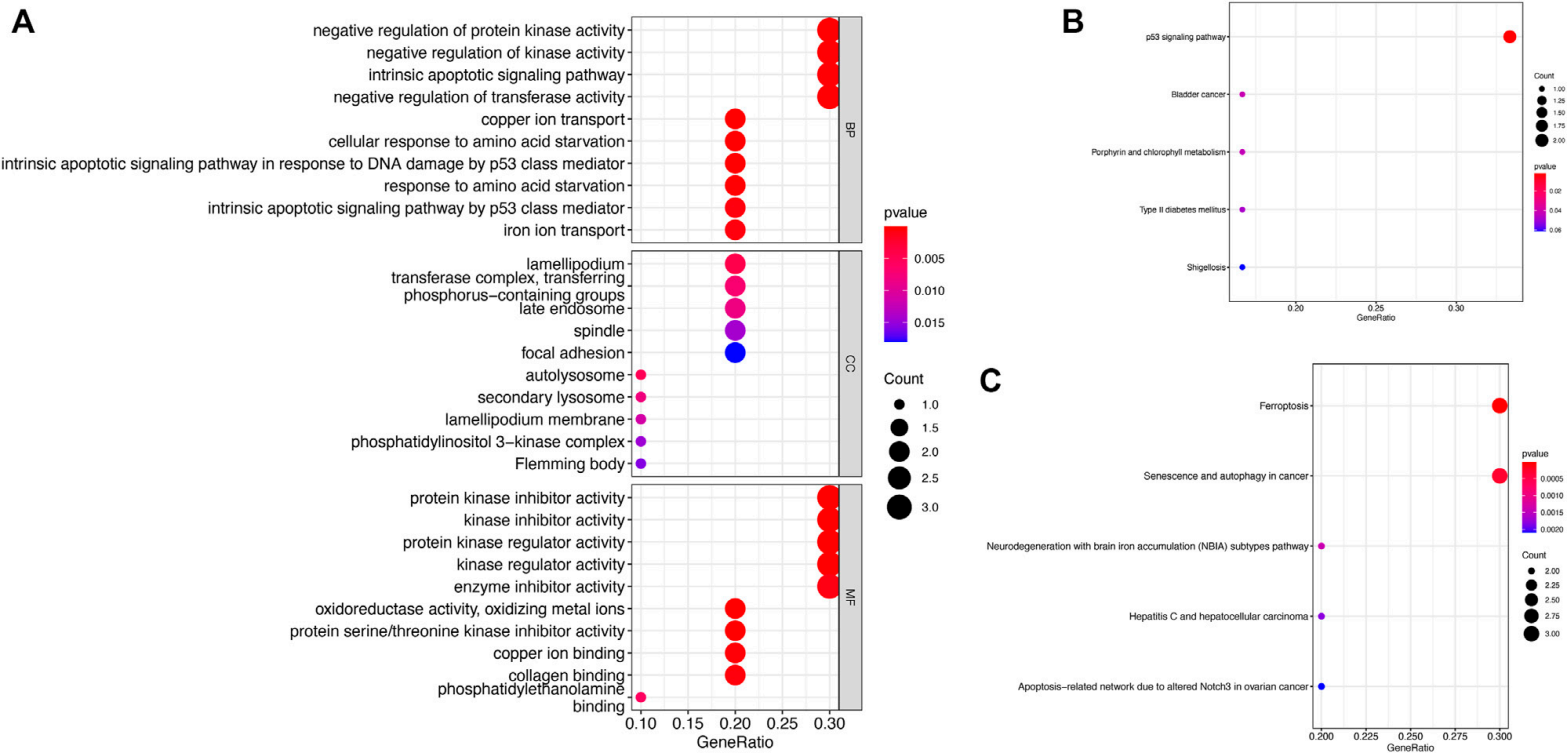
Functional enrichment analysis of key genes **(A)** GO enrichment analysis **(B)** KEGG enrichment analysis **(C)** Wikipathway enrichment analysis.

We explored the molecular mechanisms of ten key genes in GBM from the perspective of biological function, and we performed GO, KEGG, and the wikipathway enrichment analysis (Figure 11). The GO enrichment analysis consisted of three parts (biological process, cellular components, molecular function), and the bubble plots for each gene showed the top 10 significantly enriched functional items (Figure 11A). The first four significant biological process (BP) items are 1) negative regulation of protein kinase activity, 2) negative regulation of kinase activity, 3) intrinsic apoptotic signaling pathway, and 4) negative regulation of transferase activity. We also found that the first three significant cellular components (CC) items are 1) lamellipodium, 2) transferase complex, transferring phosphorus-containing groups, and 3) late endosome. The enrichment analysis showed that the first three significant molecular function (MF) items are 1) protein kinase inhibitor activity, 2) kinase inhibitor activity, and 3) protein kinase regulator activity. The most significant pathway in the KEEG enrichment analysis is the p53 signaling pathway (Figure 11B), and the top two significant pathway items in the wikipathway enrichment analysis are 1) ferroptosis, and 2) senescence and autophagy in cancer (Figure 11C).

### 3.7 Single-Nucleotide Variant Analysis

**FIGURE 12:**
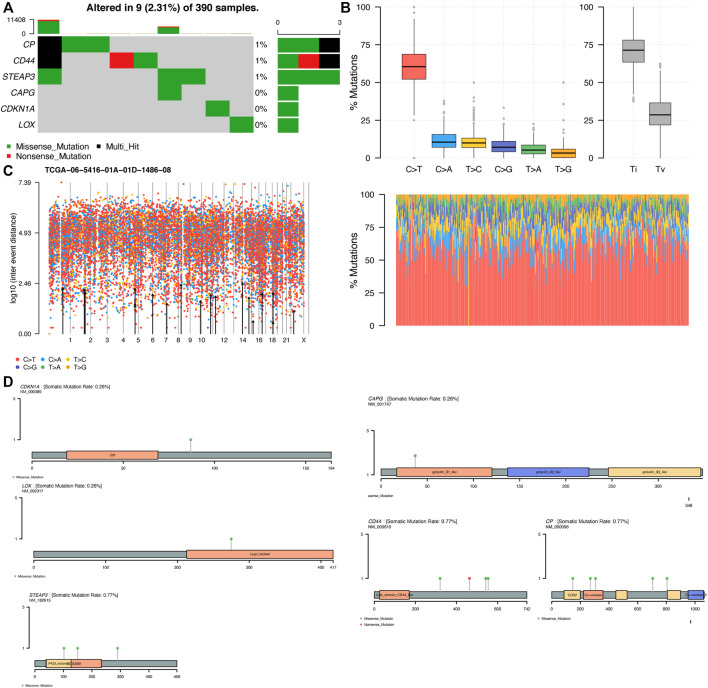
Single-nucleotide variant (SNV) analysis **(A)** The key genes are sorted according to their mutation frequency, and different colors represent different mutation types **(B)** The transition plot shows the distribution of mutations in each sample in TCGA-GBM. The stacked bar plot (bottom) shows the mutation spectrum distribution **(C)** Rainfall plot of the mutation distribution spectrum of TCGA−06−5416−01A−01D−1486–08 **(D)** Distribution of mutations and protein domain of key genes with high mutation frequency. The main body of the image shows the protein structure with the structure name marked in the box and the lollipop indicates the mutation.

We explored the molecular mechanisms of key genes from the perspective of gene mutations. The exploration of somatic mutations contributes to the understanding of tumor onset and development, and we can identify which mutations play an important role in the development of such kind of tumors, thus providing guidance on the pathogenesis and subsequent targeted treatment and prognosis of such tumors. We analyzed the SNV mutation data of ten key genes, among which six genes had different degrees of mutation (Figure 12A). Transition plots classified single-nucleotide variant (SNV) into six categories, and C > T mutations accounted for more than 50% of the total mutations among the six SNV mutations (Figure 12B). In a rainfall plot of the mutation distribution spectrum of the GBM samples, each dot indicated a mutation, and different colors of dots represented distinct SNV mutation types (Figure 12C). The mutation distribution and protein domain of key genes with higher mutation frequency were shown in Figure 12D. CP, CD44 and STEAP3 had the highest mutation frequency, and the most frequent mutation type was the missense mutation. The results showed that site mutations in these genes might play an important role in the prognosis of GBM. Mutations in these key genes are likely to make the gene replicate actively, which caused gene amplification.It is also possible that the gene will become more capable of synthesizing proteins, which will lead to high expression, resulting in a poor prognosis for the patient. Most importantly, gene amplification plays an important role in the activation of proto-oncogenes that cause cancer (George et al., 2008).

### 3.8 Correlation With Clinical Characteristics

**FIGURE 13:**
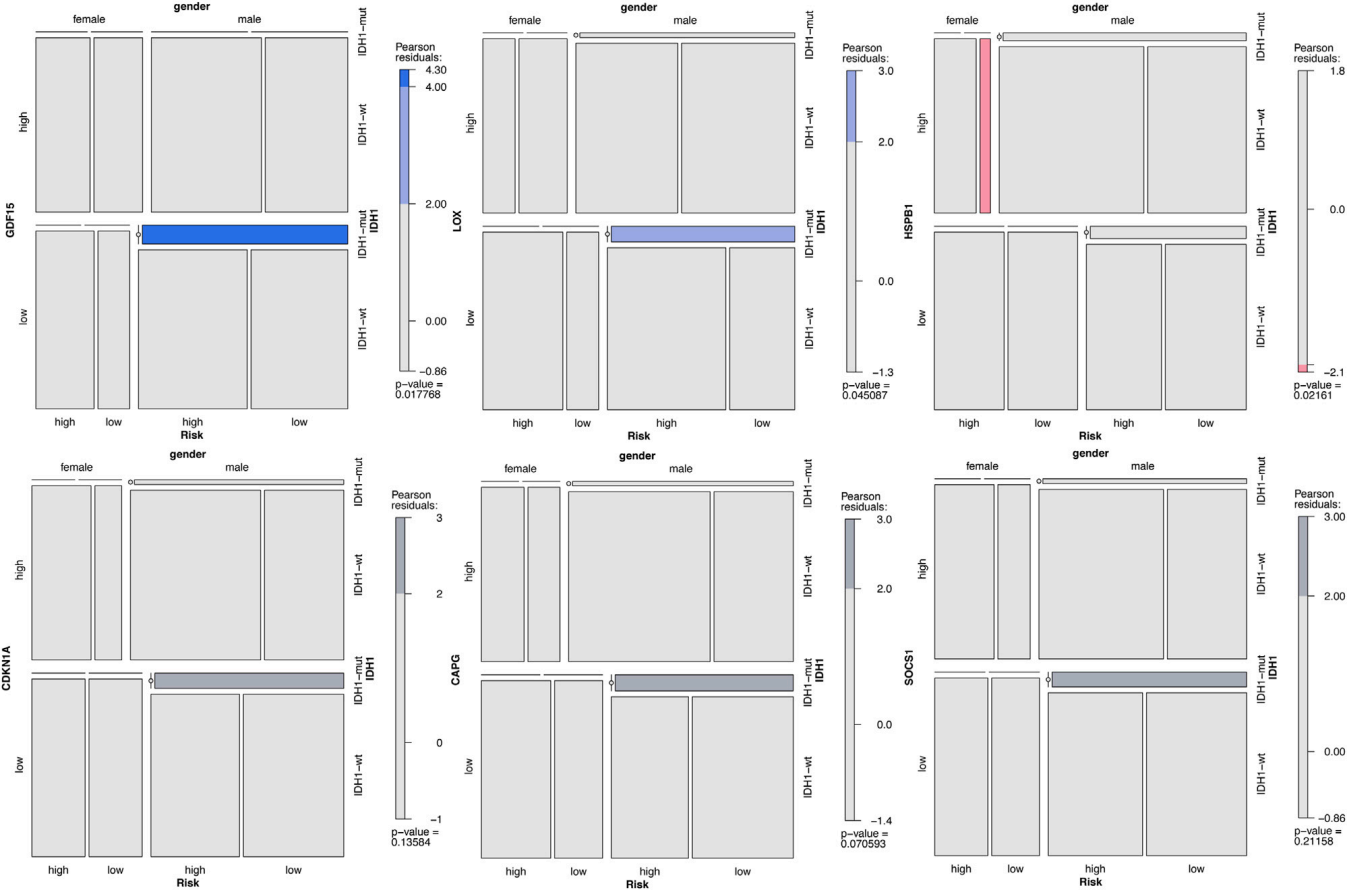
Correlation analysis between the key genes and the GBM clinical characteristics. Note: The darker the color is, the stronger the correlation is. Blue indicates positive correlation, red expresses negative correlation, and grey has a weaker correlation.

We analysed the correlation between the expression of key genes and the risk levels of different patients from a clinical perspective, and we assessed the correlation of these key genes with clinical characteristics (IDH1, gender, and risk level). We divided the expression of key genes into high- and low-level groups according to their median values. The correlation between the gene expression and the clinical characteristics was analyzed, and a mosaic for each gene was plotted. Among these ten genes, GDF15 and LOX were significantly positively correlated with IDH1 and the risk level, and HSPB1 was negatively correlated with the gender (*p* < 0.05). CDKN1A, CAPG, and SOCS1 were weakly correlated with IDH1 and the risk level (*p* > 0.05, |residuals| > 2) (Figure 13), while the remaining genes had no correlation with any clinical characteristics (Supplementary Figure S1).

### 3.9 Identification of Potential Therapeutic Drugs

**FIGURE 14:**
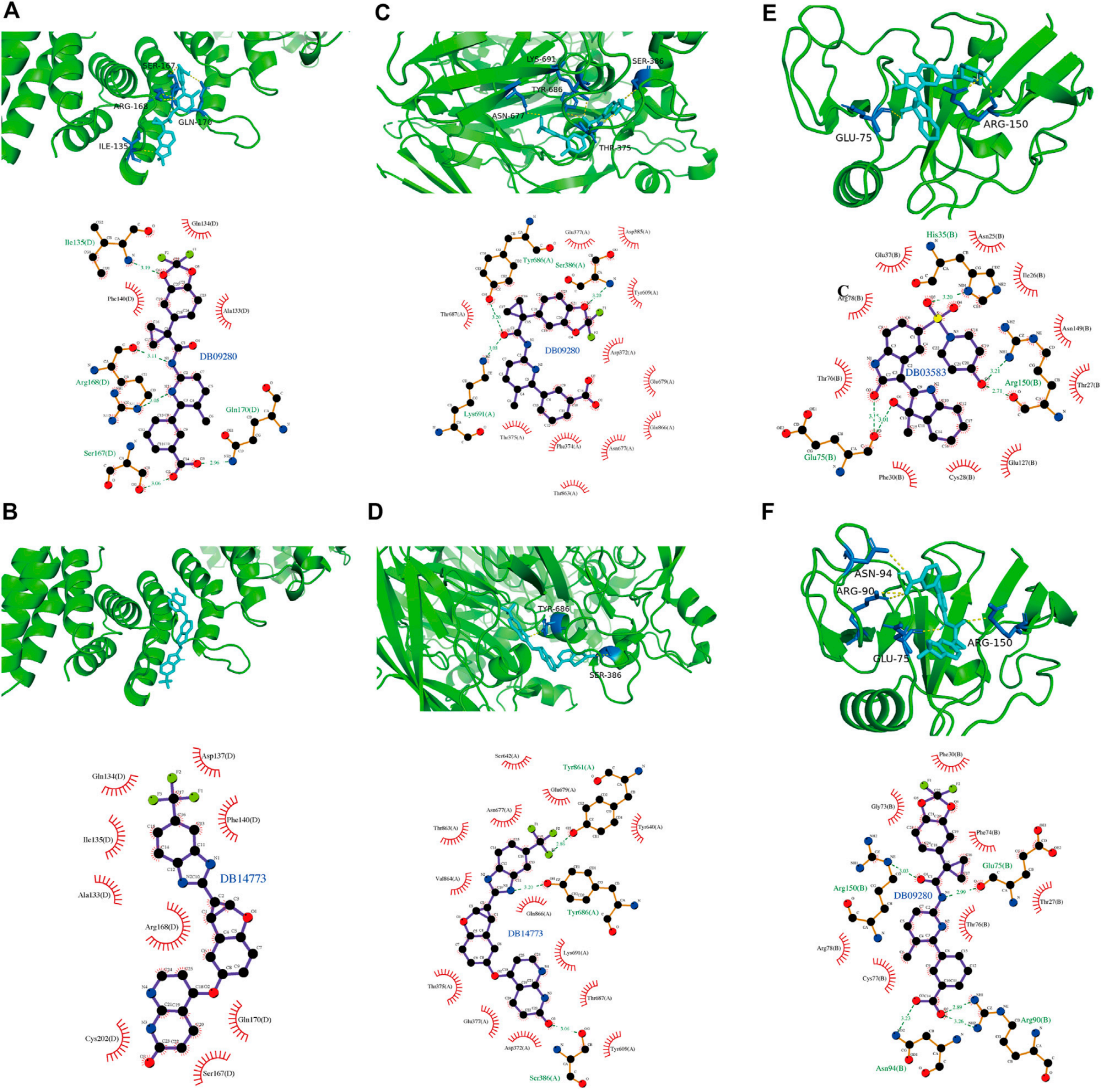
The docking conformation and interaction force analysis of CAPG, CP and CD44 with their docked compounds. Analysis of the docking conformation and interaction forces of CAPG with DB09280 and DB14773 **(A,B)**, of CP with DB09280 and DB14773 **(C,D)** and of CD44 with DB03583 and DB09280 **(E,F)**. Top half: PyMol shows docking conformation and hydrogen bonding with the color symbols of cyan for small molecule, yellow dashed line for hydrogen bonding, and blue for amino acid residues forming hydrogen bond with small molecule. Bottom half: Ligplus force analysis, small molecule in the middle, surrounded by related protein amino acid residues, green dashed line representing for hydrogen bonding formed, green amino acid names representing for amino acid residues forming hydrogen bond.

To explore potential therapeutic drugs for GBM, three genes (CAPG, CP and CD44) with more significant results were selected from the key genes based on the above analysis, and performed Virtual Screening and Molecular Docking. The top ten small molecule compounds with the best docking scores to the three key genes were shown in Table 1, and the complete table of docking scores was shown in Supplementary Table S3–S5. The docking conformation and interaction force analysis of CAPG, CP, and CD44 with the better docking compounds were shown in Figure 14. According to the Drugbank (Berman et al., 2000), The drug DB09280, which has a good docking scores to both CAPG, CP, and CD44, is the FDA (Food and Drug Administration)-approved and commercialized drug Lumacaftor (brand name: Orkambi). In addition, DB14773 has a good binding affinity to both CAPG and CP (generic name: Lifirafenib).

**TABLE 1:** Top 10 compounds with the best docking score to CAPG, CP and CD44.

Gene Name	DrugBank_ID	Hydrogen acceptors	Hydrogen donors	Rotatable bonds	LogP	Molecular weight	TPSA	Affinity (kcal/mol)
CAPG	DB09280	8	2	5	4.4	.4	97.8	−8.3
	DB14773	8	2	3	3.7	478.4	89.1	−8.2
	DB01395	3	0	0	3.5	366.5	43.4	−8.2
	DB15345	8	1	5	1.1	451.5	79.8	−8.1
	DB08683	3	1	0	3.8	393.4	65.3	−8.1
	DB06925	7	2	3	4.9	422.4	80.9	−8
	DB04760	6	2	6	3.3	410.4	84	−7.9
	DB12012	8	1	4	3.3	455.4	80.2	−7.9
	DB03571	5	3	4	2.8	430.2	127	−7.8
	DB12886	5	2	5	4.9	402.4	53.6	−7.8
CP	DB06666	3	1	3	4.8	473.9	54.3	−10.1
	DB01940	7	4	7	4.1	474.5	125	−10.1
	DB09280	8	2	5	4.4	452.4	97.8	−10
	DB14773	8	2	3	3.7	478.4	89.1	−10
	DB06075	5	2	3	4.2	421.5	89.3	−9.7
	DB07514	5	2	2	3.7	397.5	84.1	−9.7
	DB12121	6	2	4	3.6	411.5	83.4	−9.6
	DB15308	4	2	3	3.9	388.4	83.1	−9.6
	DB07794	5	2	2	1.9	327.3	97.8	−9.5
	DB00820	4	1	1	2.3	389.4	74.9	−9.5
CD44	DB03583	7	4	3	1.8	441.5	135	−8.9
	DB02354	4	3	6	3.4	423.5	97.5	−8.8
	DB09280	8	2	5	4.4	452.4	97.8	−8.4
	DB00685	10	2	3	0.3	416.4	99.8	−8.3
	DB06850	4	4	7	1.5	385.5	111	−8.2
	DB07142	5	2	4	4.9	386.5	87	−8.2
	DB05470	7	2	4	2.5	404.3	102	−8.1
	DB05608	4	1	2	2.6	400.4	102	−8.1
	DB06858	4	4	7	2.5	413.6	111	−8.1
	DB08674	5	1	0	2.8	435.5	83.2	−8.1

## 4 Discussion

In our study, 10 FRGs associated with IDH1 status and prognosis in GBM were identified using TCGA dataset, and their protein expression levels were validated. Transwell and qPCR results showed that ferroptosis promoted the migration of glioblastoma cells and affected the expression of key genes. Their biological functions were investigated by the GO, KEGG, and Wikipathway enrichment analysis. The results suggested that the key gene sets may be involved in the onset and development of GBM. These key genes with prognostic value were used to construct a prognostic model. The GSVA score was calculated for each sample using the GSVA algorithm, and the score was validated as an independent prognostic factor for GBM, and the nomogram constructed from this prognostic model had high predictive value. In addition, the analysis of immune infiltration, immunomodulators and ESTIMATE showed that the prognostic model and key genes were closely associated with immune-related factors and affected the development and prognosis of GBM. SNV analysis suggested that the mutations in the key genes might play an important role in the prognosis of GBM. Virtual Screening and Molecular Docking for potential therapeutic drugs were performed, which may provide assistance in the development of novel therapeutic chemicals for GBM.

GBM is one of the deadliest cancers worldwide. Although a great deal of research has been done in the last decade and current treatment modalities can extend the survival time and improve the quality of life to some extent, GBM remains an incurable and deadly disease, and patient survival rates are difficult to be improved (Daisy Precilla et al., 2021). Therefore, it is important to find new prognostic biomarkers and therapeutic targets for GBM.

The term ferroptosis was first coined in 2012 (Dixon et al., 2012), which means an iron-dependent regulatory cell death caused by excessive lipid peroxidation and subsequent cell membrane injury (Stockwell et al., 2017). Ferroptosis can be caused by exogenous or endogenous pathways (Tang and Kroemer, 2020). The exogenous pathway is initiated by inhibition of cell membrane transporters, such as cystine/glutamate transporters, or by activation of iron transporters, serum transferrin, and lactotransferrin. The endogenous pathway is activated by blocking intracellular antioxidant enzymes, such as glutathione peroxidase GPX4. All these mechanisms point to a common pathway, namely the formation of more reactive oxygen species (ROS). The ferroptosis pathway is a potential new target in oncology. In particular, cancer cells that are resistant to traditional therapies or have a high propensity to metastasize may be particularly susceptible to ferroptosis (Viswanathan et al., 2017; Tsoi et al., 2018), and this has opened up a new field of targeted therapeutic research.

In our study, 10 FRGs (STEAP3, HSPB1, MAP1LC3A, SOCS1, LOX, CAPG, CP, GDF15, CDKN1A, and CD44) associated with IDH1 status in GBM were identified, and survival analysis suggested that all these genes were of significant risk. Previous studies have suggested that MAP1LC3A is an autophagy-related gene and is a high-risk gene for GBM in risk modeling (Wang et al., 2019). Moreover, MAP1LC3C, which belongs to the same gene family, has significant prognostic and immunotherapeutic value in pan-cancer, especially low-grade gliomas (Zhang et al., 2022). CAPG is expressed at higher levels in glioma tissues than in normal tissues and is significantly associated with prognosis (Fu et al., 2019). SOCS1 is overexpressed in GBM and associated with chemotherapy sensitivity (Ventero et al., 2019), and the abnormal regulation of SOCS1 also enhances the resistance of GBM to ionizing radiation (Zhou et al., 2007). STEAP3 is associated with poor prognosis in GBM (Chen et al., 2021).

Ten key genes were constructed into a complex by GSVA and the GSVA score was calculated in all samples. This method is clearly different from gene signatures in other previous studies (Zhu et al., 2021), in which coefficients of genes were usually obtained from Cox regression analysis. Due to the limited sample size and tumor heterogeneity, we may never know the true coefficients of genes (Liu et al., 2020). Therefore, our study had advantages. The GSVA algorithm, a non-parametric, unsupervised method was used to score individual samples based on the key gene set (Hänzelmann et al., 2013), and numerous analysis showed that the GSVA score of the key gene set was an independent prognostic factor for OS and RFS in GBM patients. Based on this prognostic model we have also constructed a nomogram to guide clinicians in predicting the prognosis of patients and the calibration curve shows that this nomogram has a high predictive value.

Our results also revealed that the GSVA model was significantly associated with the level of immune cell infiltration, and the expression of SOCS1, STEAP3, HSPB1, CP, and LOX among the key genes was significantly associated with the level of infiltration of some immune cells. Neutrophil-triggered ferroptosis has been shown to involve in necrosis in glioblastoma and to predict poor survival (Yee et al., 2020). Our study found that the level of LOX expression was significantly and positively correlated with the level of neutrophil infiltration. In addition, we found that all key genes except for MAP1LC3A showed correlation with immunoinhibitor, immunostimulator and MHC, especially CAPG showed positive and strong correlation with all three factors. Eight key genes were known to be significantly positively correlated with stromal score and immune score by the ESTIMATE algorithm. Thus, these key genes constitute a complex that may be involved in tumor immunity and guide future therapeutic strategies for immunotherapy. Moreover, key genes with higher levels of mutation may be highly expressed as a result of gene amplification as well as affecting patient prognosis and the development and progression of GBM. A nomogram was constructed using the GSVA score to predict the prognosis of patients for clinicians, and the calibration curve showed that this nomogram had a high predictive value. The correlation of each gene expression level with the GBM clinical characteristics (IDH1, gender, and risk level) was analyzed, and GDF15, LOX, and HSPB1 were found to be significantly correlated with the clinical characteristics.

Temozolomide has a high rate of resistance as a chemotherapeutic drug for glioma, and previous studies have shown that the sensitivity of temozolomide to tumor-killing can be increased by exogenous ROS (Yin et al., 2014). It has also been shown that erastin, one of the inducers of ferroptosis, can increase the sensitivity of temozolomide chemotherapy by inhibiting the Xc-amino acid para-transport system to achieve increased ROS and induce ferroptosis (Chen et al., 2015). The results of our Transwell and qPCR experiments combined with an analysis of the available literature led us to conjecture that the drug combination might be more effective than the ferroptosis activitor erastin alone. Moreover, all key genes that we identified in our study suggested a poor prognosis for GBM patients, therefore, we selected three key genes with significant results (CAPG, CP, and CD44) for Virtual Screening and Molecular Docking, and identified three groups of small molecule compounds as potential therapeutic drugs. According to the Drugbank (Wishart et al., 2018), the drug DB09280 we identified, is the FDA-approved and commercialized drug Lumacaftor (Brand names: Orkambi), which is used for the treatment of Cystic Fibrosis (CF) in patients aged 6 years and older. The drug involved CTFR (Cystic fibrosis transmembrane conductance regulator), and it has also been shown that the activation of the CFTR involved in this drug inhibits the proliferation, migration and invasion of GBM cells by suppressing JAK2/STAT3 signaling (Zhong et al., 2019). However, according to our results, the drug may be able to treat GBM through the pathway of CAPG, CP and CD44, and further studies *in vivo* will be needed.

In addition, the functional enrichment analysis showed that the key gene set was significantly involved in the p53 signaling pathway, senescence and autophagy in cancer, and in the negative regulation of protein kinase activity. Previous studies have suggested that the p53 protein and its cellular pathways mediate tumor suppression through an informed, regulated, and integrated response to the environmental perturbations that lead to cell death or maintain cellular homeostasis (Levine, 2020). Inactivation of TP53 (Tumor Protein p53) is the most common mutation in sporadic human cancers because the TP53 gene encodes a transcription factor that is an important barrier to carcinogenesis, which also suggests a strong correlation with p53 function during tumorigenesis and may be associated with the occurrence of the glioma (Kandoth et al., 2013). Autophagy is a lysosomal degradation process that is critical for cellular homeostasis and adaptation to stress. There is growing evidence that autophagy declines with age. The individuals with impaired autophagy are susceptible to age-related diseases, and the stimulation interventions of autophagy tend to promote longevity (Leidal et al., 2018). It is suggested that the key genes may be involved in the onset and development of GBM through these pathways. However, further studies are needed to investigate and validate the functions of these genes.

## 5 Conclusion

In conclusion, our study established a ferroptosis-related prognostic model for GBM patients based on the screened ten key genes by a different modeling method from previous study, the GSVA algorithm. The nomogram was also established to assist clinicians in decision-making. The molecular mechanisms were investigated by several methods including cell biology experiments, functional enrichment analysis, immune cell infiltration analysis, immune-related factors analysis, ESTIMATE and SNV analysis. With the support of these evidences, the key gene set might be involved in the development and onset of GBM. Three groups of potential therapeutic drugs were identified through Virtual Screening and Molecular Docking. These results bring light to the diagnosis and therapy of GBM.

## Data Availability

The original contributions presented in the study are included in the article/Supplementary Material, further inquiries can be directed to the corresponding authors.
