# Supplementary figures and images for: Modulation of Tumor Immune Microenvironment and Prognostic Value of Ferroptosis-Related Genes, and Candidate Target Drugs in Glioblastoma Multiforme
Source: Front Pharmacol. 2022 Apr 28;13:898679. doi: 10.3389/fphar.2022.898679 (PMC9095828; doi:10.3389/fphar.2022.898679)

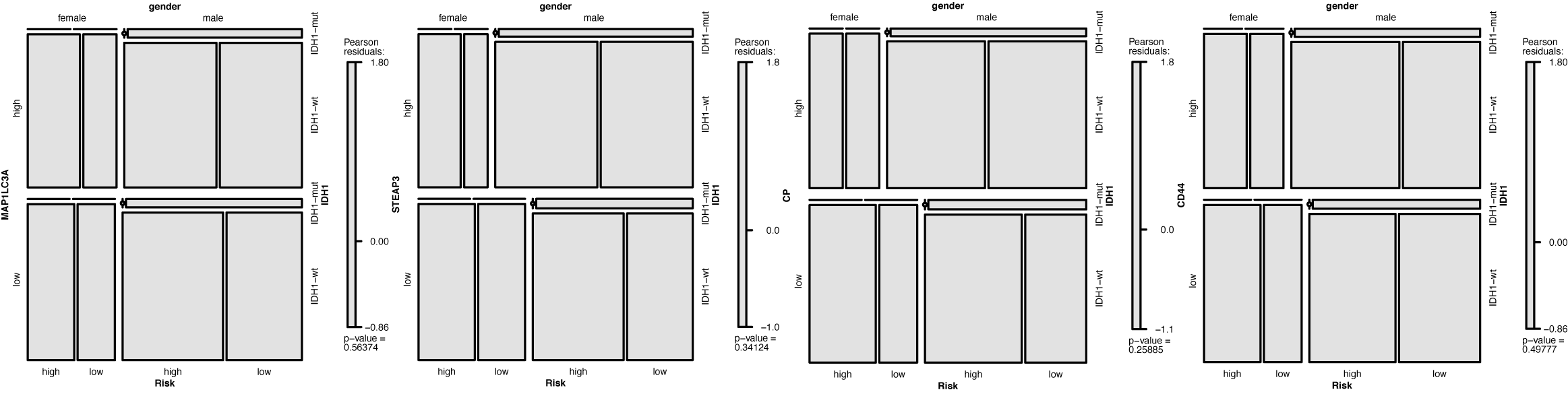

Supplement: Supplementary file 5 [file Image1.PNG]
